# Advances in Cruciform Biaxial Testing of Fibre-Reinforced Polymers

**DOI:** 10.3390/polym14040686

**Published:** 2022-02-11

**Authors:** Sergio Horta Muñoz, María del Carmen Serna Moreno

**Affiliations:** Escuela de Ingeniería Industrial y Aeroespacial de Toledo, Instituto de Investigación Aplicada a la Industria Aeronáutica, Universidad de Castilla-La Mancha, Av. Carlos III, Real Fábrica de Armas, 45004 Toledo, Spain; mariacarmen.serna@uclm.es

**Keywords:** biaxial loading, fibre-reinforced polymer, cruciform specimen, tensile/compressive loading, failure theories, multiaxial, testing facility, finite element method

## Abstract

The heterogeneity and anisotropy of fibre-reinforced polymer matrix composites results in a highly complex mechanical response and failure under multiaxial loading states. Among the different biaxial testing techniques, tests with cruciform specimens have been a preferred option, although nowadays, they continue to raise a lack of consensus. It is therefore necessary to review the state of the art of this testing methodology applied to fibre-reinforced polymers. In this context, aspects such as the specific constituents, the geometric design of the specimen or the application of different tensile/compressive load ratios must be analysed in detail before being able to establish a suitable testing procedure. In addition, the most significant results obtained in terms of the analytical, numerical and experimental analyses of the biaxial tests with cruciform specimens are collected. Finally, significant modifications proposed in literature are detailed, which can lead to variants or adaptations of the tests with cruciform specimens, increasing their scope.

## 1. Introduction

The large increase in the structural use of fibre-reinforced polymer (FRP) composites has generated great scientific interest in the study of its mechanical behaviour. They are a versatile category of materials, including different dispositions and a range of fibre reinforcement, supporting matrices and arrangements of plies, whose combination provide flexibility in manufacturing and possibilities of optimisation in the design of structural components. In terms of mechanical properties, their high strength and low density have made them a preferred option in sectors such as aerospace, sports or wind energy, in which structural efficiency is a priority [1].

Proper laboratory testing forms the basis for determining the mechanical response of FRP. Many of the experimental methods have their origin in the testing of metals for which, due to their significant historical development, there are more types of testing than for the relatively new FRP. However, these tests often do not meet the particular requirements of FRP because composites have an anisotropy and heterogeneity, which are not so significant in metals. The strength and stiffness in the different directions of the FRP can differ by even two orders of magnitude. The choice of an inappropriate testing methodology can also lead to the determination of characteristic values with high dispersions.

Although for the uniaxial case, there are regulations for the characterisation of most materials, and in particular for the FRPs, it is currently not possible to find a standard for testing FRP under external loading in multiple directions. Furthermore, from a scientific point of view, the damage models and failure criteria of composite materials continue to pose certain uncertainties. In the absence of failure criteria applied to composite materials, Hinton, Soden and Kaddour promoted in 1991 the initiative of the first world-wide failure exercise (WWFE-I). This scientific collaboration aimed to create theoretical predictions for the damage and failure of composites, which were simultaneously validated with experimental data obtained from multiaxial testing [2,3,4,5]. Analysing certain laminate configurations established by Hinton and Soden [2], recommendations were obtained for the design and multiaxial characterisation of these materials [5]. The relevance of the topic of multiaxial damage and failure in FRP composites led the promoters of this exercise to extend it into two more stages, the WWFE-II and WWFE-III [1,6,7,8,9,10,11].

Among the conclusions of the WWFE-I, Soden et al. [5] emphasise that, despite obtaining a large amount of experimental data, they found a limited number of studies in which a wide range of different loading ratios were achieved in specific laminates. In addition, most of WWFE experimental results were obtained with tubular specimens, which present difficulties as obtaining a repetitive geometry or determining the initial failure. Therefore, while numerous failure criteria have been developed analytically and numerically validated, to this day it is still difficult to find experiments that reproduce multiaxial stress states, either biaxial or triaxial. This is because these tests are time consuming, costly, difficult to perform, and their results are complex to post process [12].

Structures in aerospace and wind energy industries using FRP composites, mainly carbon (CFRP) and glass fibre reinforced polymers (GFRP), are frequently characterised by small thicknesses and submitted to multidirectional loads. Nevertheless, these structures can be approached by a plane stress simplification, where the significant stresses are the two normal and the in-plane shear components (i.e., out-of-plane components of the stress tensor are negligible). Olsson [13] carried out a review of the types of tests that generate multiaxial states in order to measure strength in laminates, both in-plane and out-of-plane. In the case of planar biaxial states, tests with cruciform and tubular specimens are described, which allow generating any plane stress state, as well as other methodologies that permit to achieve specific biaxial stress/strain states.

A similar classification of multiaxiality was proposed by Quaresimin and Carraro [14], in which the authors spoke of external multiaxiality as that achieved when loads are applied in several directions (i.e., tests with tubular and cruciform specimens), while internal multiaxiality appears when the fibres form an angle with the loading directions. For instance, the well-known uniaxial off-axis test makes possible to achieve certain in-plane biaxial stress/strain states, depending on the angle formed between the load and the fibres, although it is not feasible to achieve every possible biaxial state [12,15,16]. Furthermore, the results obtained with off-axis tests were proved by Cai et al. [17] not to be as representative as those obtained with other biaxial testing techniques.

Focusing on external multiaxial tests, Smits et al. [16] pointed out how tests with tubular specimens may lead to certain inconveniences. Listing a number of these drawbacks, they are as follows: the presence of radial stresses could be not negligible, the properties of plane plates and tube shells are not directly comparable (e.g., this could be easily related to the curvature of fibres), and specimens in the form of thin-walled tubes can experience instability phenomena when subjected to compression or torsion. These tubular specimens require a certain combination of tensile (by means of internal pressurisation), twisting loads and stacking sequences to reach different biaxial loading ratios [14], while compression may be achieved by means of external pressurisation, which is difficult to obtain in practice [12]. Currently, this type of test is postulated as a suitable solution in multiaxial fatigue tests [14,18,19,20].

Following the WWFEs studies, cruciform specimens are used more frequently in biaxial testing. Although biaxial testing has been performed for many years [21,22,23], in the last two decades, many improvements have been extensively proposed to assess multiaxial mechanical characteristics. For instance, Smits et al. [24] made a short review of the biaxial tests on cruciform specimens carried out up to 2007, probing the existence of a scientific community involved in the development of the biaxial characterisation of FRP, but concluding with the need for further research in this domain. The existence of a test methodology which establishes the geometry and dimensions of the specimen, the properties to be measured and even the equipment and instrumentation to be applied would reduce the uncertainty. In addition, it would simplify the process of characterising the multiaxial response of the material to make it more attractive at the level of development and design of structures. This review will address the different approaches given to these issues in recent years, highlighting the solutions achieved and the aspects that continue to raise debate.

## 2. Testing Facility: Advances in Testing Control and Data Acquisition

Biaxial testing with cruciform specimens consists in the direct application on the arms of the sample of tensile and/or compressive loads in the two in-plane perpendicular directions so that it is possible to obtain the desired ratio of biaxial stress/strain, acting on the applied force/displacement in both directions. Even though the concept of biaxial testing with cruciform specimen is simple to elucidate, the design of the testing facility and the specimen is highly complex [25]. In this section, some of the solutions achieved relating testing equipments are discussed, as well as recommendations to take into account and possible aspects of future improvement in the issues related to the testing methodology.

There is a certain synergy in the application of this experimental technique to different categories of materials, since the test with metallic alloys is the main source of progress. However, certain specific characteristics of composite materials, mainly due to their heterogeneity and anisotropy, make it such that any extrapolation of the methodologies applied to other materials must be rigorously analysed.

Some of the first designs of experimental facilities for biaxial characterisation using cruciform specimens were developed more than 50 years ago [21], achieving significant advances in the last decades of the 20th century [26,27,28]. Despite this long history, currently biaxial testing machines are still uncommon in laboratories for the basic characterisation of mechanical properties, although it is possible to find commercial equipment, generally ad hoc designs [29,30,31]. These biaxial machines are based on the design by Welsh and Adams [25], but consist of four actuators (hydraulic or electromechanical) faced by pairs in the same plane.

It should be noted that a biaxial testing machine is a complex installation. Firstly, it should be highlighted the cost and space necessary to apply tensile and compressive loads in a controlled way in two perpendicular directions, added to the possible need to carry out measurements in those two load directions. Secondly, a control scheme is required to avoid the appearance of deflections or undesired shear loads, for which regulation schemes through closed loop and control of the midpoint displacement [30,32,33] are frequently utilised.

### 2.1. Load Frame

The collaboration initiated by Welsh and Adams from the “Air Force Research Laboratory” and the University of Wyoming [25,34,35,36,37] was one of the pioneers in conducting biaxial tests with cruciform specimens of FRP composites. These authors used an electromechanical triaxial machine, whose development is detailed in [25]. The researchers describe the ability to apply uni-, bi- and triaxial loads on cruciform specimens, by means of pairs of facing 94 kN actuators disposed in three orthogonal directions. In this work, the measurement of the compliance of the machine and the electronic programming of the control scheme and data acquisition are emphasised. It is worth highlighting the development of a tool that allows to ensure the alignment of the specimen with the load directions, a critical parameter to ensure the desired state of stress. Regarding the regulation control, the preferred solution was a master–slave scheme, in which a pair of actuators (masters) receive the set displacement/force rate, while the rest (slaves) try to maintain a ratio with respect to the master. In this work, the authors noted the capacity to maintain stress ratios within 2% of the desired value.

**Figure 1:**
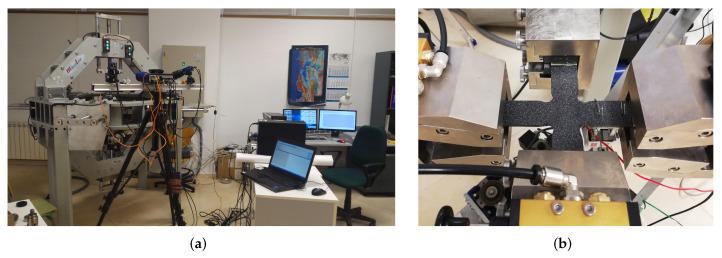
Triaxial testing facility at University of Castilla-La Mancha (UCLM, Spain): (**a**) triaxial testing machine. (**b**) CFRP cruciform specimen being tested under biaxial tensile–tensile loading.

An installation very similar to the previous one was used in the works of Serna Moreno et al. [38,39,40], which is shown in Figure 1. This facility consists of six electromechanical actuators capable of providing up to 50 kN, both tensile and compressive, maintaining a certain load or displacement rate in each direction. Similarly, Makinde et al. [26] employed a machine with 250 kN hydraulic actuators in which opposing actuators shared the same oil supply to ensure that the forces exerted were equivalent. As a summary, the equipment with the best acceptance in the scientific community follows this same scheme of two orthogonal pairs of axially aligned actuators with independent load cells, whether they are electromechanical or hydraulic. Examples of similar facilities are found in [16,17,41,42,43,44,45,46,47].

Hannon and Tiernan [48] reviewed the in-plane biaxial systems applied to the characterisation of sheet metal because biaxial states can be present in sheet metal forming processes and the interest of the biaxial response due to the anisotropy resulting in the material after manufacturing. The specimen design included in this study is not described here, as these present particularities related to the material do not make them completely suitable for the testing of composite materials. This is explained due to the ductility of metals promoted by plastic behaviour, which contrasts with the inherent brittleness of composites. However, the testing machines would be similar to those applicable in FRP. In this review, the testing systems are classified in two broad categories: stand-alone biaxial testing machines and link mechanism attachments. The facilities described so far belong to the first family. However, the high cost of this type of machinery has led to the design of different link mechanisms that allow adapting universal uniaxial machines for the application of certain force ratios, although it was found that most of these designs have numerous drawbacks.

Ferron and Makinde [28] engineered one of the earliest design of this kind of tools. This device is based on a symmetrical mechanism formed by joint arms that enable different force/displacement ratios between the orthogonal directions. The value of these ratios depends on the lengths of the arms, achieving equal or unequal stretching ratios, obtaining even a case of plane strain by using rigid arms instead of articulated ones. It should be noted that this type of fixture neither allows the combination of tensile and compressive loads to be introduced, nor permits to control the applied stress or the variation of the applied deformation ratio during testing. However, this type of equipment is still developed, finding numerous works in the literature, designing and applying similar systems [47,49,50,51,52,53,54,55]. As a matter of example, Puente et al. [54] carried out a comparative study of five of these devices, predicting their dynamic response through simulations based on the finite element method (FEM). This is also validated through the manufacturing of scale prototypes using fused deposition modelling (FDM). Through the combination of simulations and manufactured models, it is verified that these devices present unsuitable movements, or they are limited in their range of use.

Another notable development in biaxial testing systems, although intended for metals, is described in the work of Merklein and Biasutti [56]. The researchers designed an electromechanical facility, based on the work by Geiger et al. [57], which aims to combine the advantages of both families of biaxial machines (stand-alone facilities and link mechanism devices). While the cost is reduced, compared to a fully stand-alone facility by avoiding the use of several servo-controlled actuators, this configuration with a single motor allows the application of different load ratios by varying the angle of the transmission bars, without altering their geometry. The use of two load cells permits the measurement of force in both directions, and the ability to generate equibiaxial and non-equibiaxial stress states is experimentally demonstrated.

### 2.2. Strain Monitoring and Controlling Techniques

Regarding the data acquisition during testing, the estimation of the stress/strain state in the biaxially loaded gauge zone relies on strain measurement. Some pioneering biaxial studies were based on techniques such as photoelasticity [58] or the use of displacement transducers and axial extensometers [26]. For instance, Makinde et al. [27] proposed the design of a biaxial extensometer for the measurement of strains in the two in-plane perpendicular directions at the gauge zone of cruciform specimens, which could register even triaxial strain states. Although this methodology has not been used in subsequent studies considering biaxial testing, it served as the basis for useful measurement techniques in other types of tests, such as the triaxial test proposed in the work by Hayhurst and Felce [59].

The work of Ramault et al. [60] synthesises this topic related to biaxial tests. The investigators belong to a group of researchers from the Vrije Universiteit Brussel and Ghent University who carried out numerous biaxial studies on GFRP [16,24,61,62,63,64,65,66,67,68,69,70,71]. In [60], the authors experimentally compared three strain measurement techniques, reviewing their advantages and disadvantages. Specifically, strain gauges, electronic speckle pattern interferometry (ESPI) and digital image correlation (DIC) are applied to perform uniaxial and biaxial tests of a GFRP laminate sequence of interest for wind turbine blades. To do this, a geometry of the specimen [16] previously analysed by FEM is used in a testing machine that consists of four servohydraulic actuators with a force-controlled closed-loop system. With regard to strain gauges, their high precision contrasts with their capacity to measure only local results and the possibility of premature debonding if the material withstands large strains. Although ESPI offers the advantage of a full-field measuring technique, this study points out as a drawback the fact of oversensitivity to the presence of vibrations generated by the biaxial testing machine. Finally, the DIC technique also permits displacement and strain full-field measurement, thanks to the correlation of images captured at different moments of the test, having previously applied a black and white speckle pattern to the specimen. This system does not interfere with the test, as it is a non-contact technique, although it presents more deviation in the results and may fail to correlate displacements when discontinuities occur.

The referenced literature highlights the predominant use of strain gauges [17,36,37,38,39,40,41,46,58,72,73,74,75,76,77,78,79,80,81,81] which provides simplicity when recording the strain state in the gauge zone, in the absence of large strain gradients, such as open hole concentrators [60]. Another noteworthy advantage of the strain gauge technology is the need of a small space for its placement and wiring, which makes it an ideal technique for tests in which access to the specimen is limited [58,74,82]. An application that demonstrates the versatility of strain gauges is the ability to adhere them at both (top and bottom) surfaces of the specimen in such a way that they facilitate finding the bifurcation point caused by buckling by registering opposite trends in the measurement of strains resulting from the curvature of the specimen [45,75,76,78].

Finally, strain gauges can be applied for the calibration of biaxial test equipment. On the one hand, Kwon et al. [50] used a strain gauged dog-bone specimen in order to detect deviations in the biaxial testing mechanism mounted on a uniaxial machine. On the other hand, the company MTS [30] provides a commercial solution to check the alignment by means of a cruciform specimen instrumented with several strain gauges. The objective of this type of instrumented calibration specimens is to reduce the appearance of bending caused by the application of loads misaligned with regard to the in-plane orthogonal directions.

However, there is a consensus in the research field to prefer the use of full-field techniques, emphasising the case of DIC, which has been applied, to date, in numerous biaxial tests with cruciform specimens [44,46,60,61,65,67,69,71,78,81,83,84,85,86,87,88]. In addition to the detailed visualisation of the strain contours in different directions at every point on the surface, this technique provides numerous advantages. Among them, the ability to evaluate the homogeneity of the strain fields stands out [46,60,89].

Several researchers have been able to take advantage of DIC to provide new approaches to the characterisation of material properties through biaxial tests. In the works of Périé et al. [88,90], the acquired displacement fields are used as input data in a FEM analysis to obtain a damage law of an anisotropic material. Similarly, Lecompte et al. [91] estimated the orthotropic elastic properties of a GFRP lamina by using an inverse FEM technique, minimising the difference between the strains obtained experimentally and numerically. Other studies following a similar scheme are the ones carried out by Schemmann and Lang et al. [32,33,92]. These works are among the few that focus on studying unreinforced and continuous fibre-reinforced thermoplastic polymers. In particular, the Reference [92] detailed the inverse parameter identification in a long glass fibre-reinforced polypropylene. The strain fields obtained by DIC are compared with the numerical results from FEM simulations, and an optimisation algorithm is applied to minimise the difference between these fields obtaining the anisotropic viscoelastic properties of the material. These properties are later homogenised by means of common mean field homogenisation techniques, such as the interaction direct derivative estimate and Mori-Tanaka.

Another interesting development of the DIC techniques was performed by Busca et al. [93]. In order to characterise the fatigue behaviour of composite materials under multiaxial loadings, the cyclic tests are monitored simultaneously with a system of two high-speed cameras combined with infrared thermography. The objective of using the high-speed stereo DIC system is to quantify local stiffness degradation, while temperature fields allow a qualitative assessment of damage.

Additionally, the use of 3D DIC technology also helps in measuring the displacements produced in the direction perpendicular to the plane, which allows evaluating the presence of misalignments or the appearance of buckling under compressive loads [78]. Finally, it should be noted that this technology is not only used for test monitoring, but can also intervene in testing control. Moncy et al. [46,94,95] highlighted the need to carry out strain control, which produces more adequate results than methodologies based on force or displacement control when studying crack growth. However, strain control requires taking into account material stiffness degradation. To do this, these authors proposed a real-time digital image point tracking system, which allows strain control to be carried out by means of closed-loop regulation. They take into account strains recorded on live at the central region of the specimen by means of DIC cameras, in a similar way to a video extensometer.

## 3. Specimen Design

In addition to the complexity of the testing facility presented in the previous section, specimen design is addressed in the majority of literature for developing cruciform biaxial testing. This issue poses the major challenge of achieving a uniform biaxial stress/strain state in a sufficiently large area, while stress concentrations appear at the corners between adjacent arms, which may promote undesirable failure. This is added to the fact of introducing simultaneous tensile and compressive loads with different ratios and even in a cyclic manner for fatigue study. The practically inevitable presence of concentrations leads most of the authors to perform thickness tapering. In spite of this, it is concluded that at certain load states, it is concluded that the cruciform specimen test is not suitable as a strength determination test, but rather for the evaluation of the mechanical response prior to final failure [46,78].

Bearing in mind that this type of test is only standardised in the case of its application to metallic materials [96], any test proposal on unreinforced or reinforced polymers must be accompanied by the design or selection of a specimen. This section describes the work done by numerous authors to solve problems in this aspect.

Numerous alternatives have been raised mainly in the past two decades, usually opting to analyse the geometry through numerical simulations based on FEM. These studies may be initially classified in two groups: studies that try to devise geometries with parametric variations of certain dimensions and geometrical features [16,63,64,97], and works focused on shape optimisation [65,98,99]. In any case, the geometries resulting from these studies share similar characteristics that are discussed throughout this section.

Previously, some main characteristics of the biaxial test were presented, which can be summarised in those collected in one of the pioneering works on biaxial characterisation of composites [16]. Smits et al. proposed five requirements that the design of the specimen must accomplish, which are reflected in a similar way in several of the following studies:

- Maximise the homogeneity of stresses and strains in the region of interest (ROI), that is, in the biaxially loaded zone.
- Minimise the undesired in-plane shear stress/strains in the ROI. To this requirement, it can be added to avoid the appearance of out-of-plane displacements due to geometrical imperfections of the specimen, as well as those due to instabilities under compressive loadings.
- Achieve failure in the gauge zone. It should be taken into account that this requirement is given in those works that aim to strength characterisation, as well as those that propose the verification of failure theories (for example, works related to the WWFE). However, many researchers focus their biaxial studies on aspects other than failure, but they generally share the need to postpone failure outside the biaxial zone as much as possible, for instance, in order to record most of the mechanical response under biaxial load (including plasticity, damage, etc.).
- Repeatable and reproducible results.

More requirements are added to this list by other authors, such as the ability to obtain a wide range of stress/strain ratios in the central zone, including states combining tension and compression in all their possibilities [33].

However, after decades of research in cruciform specimens with different families of materials, works finding areas for improvement continue to appear. One of the concerns in the case of biaxial tests is the quantification of the force (or stress) that reaches the central zone. In this sense, Welsh [37] defined a bypass correction factor, obtained through the measurement of strain in an uniaxially loaded cruciform specimen and taking into account the elastic modulus of the laminate calculated by means of the classical laminated plate theory. A similar strategy defining a factor relating the applied force and the central stress is employed by other authors. For instance, Serna Moreno and López Cela [38] defined a factor, which is dependent on the specimen geometry and force ratio applied, that is computed from numerical simulation. Cai et al. [100] agreed that the stress coefficient depends on the specimen geometry, thickness ratio between the central zone and the arms, and the applied loading ratio. The drawback of this solution is that factors are obtained by means of linear elastic analysis, so the range of application is limited until the appearance of material non-linearities. A similar result was reached in the work of Van Hemelrijck [70], where stresses were obtained from the measurement of strains in combination with the constitutive equations of the material, which is also limited to the elastic behaviour.

Moncy [46] analysed some typical geometries and quantified the amount of load sharing between adjacent arms, verifying numerically how the geometry of the central zone significantly affects the amount of applied force needed to reach a certain level of strain in the gauge region. Furthermore, this author concluded that slotted arms maximise the applied force reaching the central zone.

A more detailed approach to the analysis of loads reaching the gauge zone was carried out by Nolan and McGarry [101]. In this work, they stated, based on analytical developments and numerical simulations, that the measurement of forces applied by the actuators does not allow to estimate the stresses developed in the biaxial zone, due to the lack of estimation of shear forces in the clamps. Therefore, in the words of the authors, the use of force correction factors for stress estimation in the biaxially loaded zone is highly inaccurate. Then, they proposed to rely on inverse FEM analysis, although these entail the need of previously knowing the material constitutive model. However, it should be noted that these factors are imprecise only if they are used uniformly for different applied loading ratios and/or cruciform specimen geometries.

Continuing with the analysis of the force that crosses the gauge zone, and adding the requirement to maximise the stresses developed in the biaxially loaded zone, most studies agree on the need to act on the geometrical features and dimensions of the central zone of the test specimen. In this sense, two main lines of action can be defined: soften the stresses in the regions surrounding the biaxially loaded zone (i.e., arms and corners), or increase the stress produced in the central region by reducing the thickness. These two approaches are detailed in next subsections.

### 3.1. Mitigation of Stress Concentrations outside the Gauge Region

First of all, it is important to note that most cruciform specimens have filleted corners between arms, as these are an important stress concentrator that promotes premature failure and can distort the stress/strain field obtained in the ROI. In addition, another possibility that allows maximising the stresses in the gauge zone is the machining of slots in the arms of the specimen. It should be noted that this option has traditionally been applied mainly in biaxial studies with metals [102,103], due to the difficulty in machining composite materials, that usually results in delamination [46]. In fact, the standard for biaxial characterisation of metal sheets [96] establishes the presence of slots in the arms of the standardised design of the cruciform specimen.

**Figure 2:**
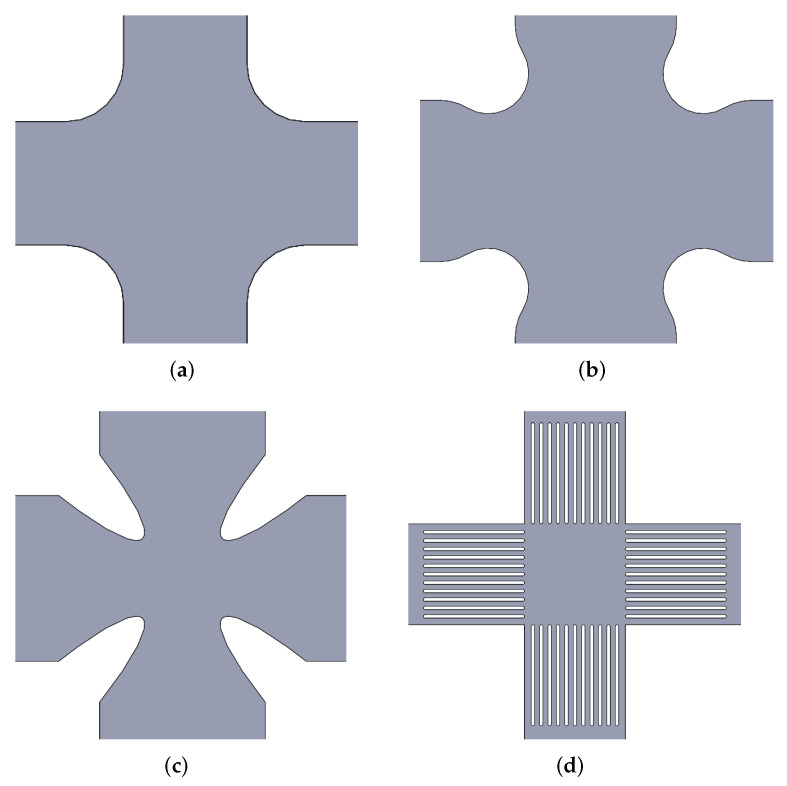
Schematisation of the most frequently used geometrical features in cruciform specimens: (**a**) Single radius corner. (**b**) Double radii filleted corner. (**c**) Elliptical corner. (**d**) Slotted arms.

The geometry and dimensions of these filleted corners and slots have been discussed in numerous works, and there is still no clear consensus on the most appropriate option, so this review summarises the preferred choices by different researchers in Table 1. For a better understanding of these designs, Figure 2 outlines those options frequently exposed in the literature.

### 3.2. Maximisation of Stresses in the Gauge Region

Regarding the approach based on thickness reduction, it should be noted that it has also been the topic of numerous investigations, including both numerical simulations and experimental tests. Despite that the literature usually recommends thickness tapering, two different approaches are proposed to implement the change in thickness. On the one hand, some authors suggest keeping the thickness constant throughout the specimen to be tested, and reinforcing both the arms and the area near the corners between arms with tabs (generally made of composite material or aluminium). In this way, the material to be tested must only be machined in its outer contour, and the tabs are machined in their central area, leaving the gauge zone accessible for measuring. This option is firstly described in the works by Kumazawa and Huang et al. [42,81,120], and similarly reproduced in subsequent works by other authors [44,83,85,87,104].

Most of these works obtain the specimen by adhering the machined tabs on the contoured specimen, but Escárpita et al. [104] performed a modification in the manufacturing methodology. The authors chose a non-impregnated fabric for the outer reinforcing layers, and therefore both specimen and reinforcing layers require to be resin impregnated and cured. Consequently, the dry fibre layers used for tabbing are cut and placed using hand layup under and over the central layers, and the whole laminate is impregnated and cured in a single step. Torres and Maji [111] carried out a biaxial study on the effect of open-hole concentrators in woven carbon/epoxy under tensile–tensile biaxial loading. Regarding the design of the specimen, two methods of tabbing were proposed. Type-I specimen, as named by the authors, includes glued aluminium tabs comprising only the region at the corner between arms. Meanwhile, the Type-II specimen consists of a composite tab covering the arms and the corners between them, while leaving untabbed a large squared centre region. The first type failed prematurely, observing lower maximum stresses than other options, and a failure mode inappropriate for strength characterisation (i.e., failure is initiated outside the gauge region). Kobeissi et al. [85] defended the use of aluminium tabs versus composite ones, due to the facility of machining metal, the risk of delamination on composite tabs and the capacity of withstanding larger strains, thanks to metal plasticity. Moreover, authors validated their design through numerical simulations based on FEM, including cohesive elements representing the adhesive layer between the composite specimen and the aluminium tab. These analyses account for the effect of the specific aluminium alloy chosen as the tabbing material, the thickness of the tab and the shape of the thickness transition (discussed below). The numerical study is experimentally validated, performing tests under different states of tensile–tensile stress.

**Figure 3:**
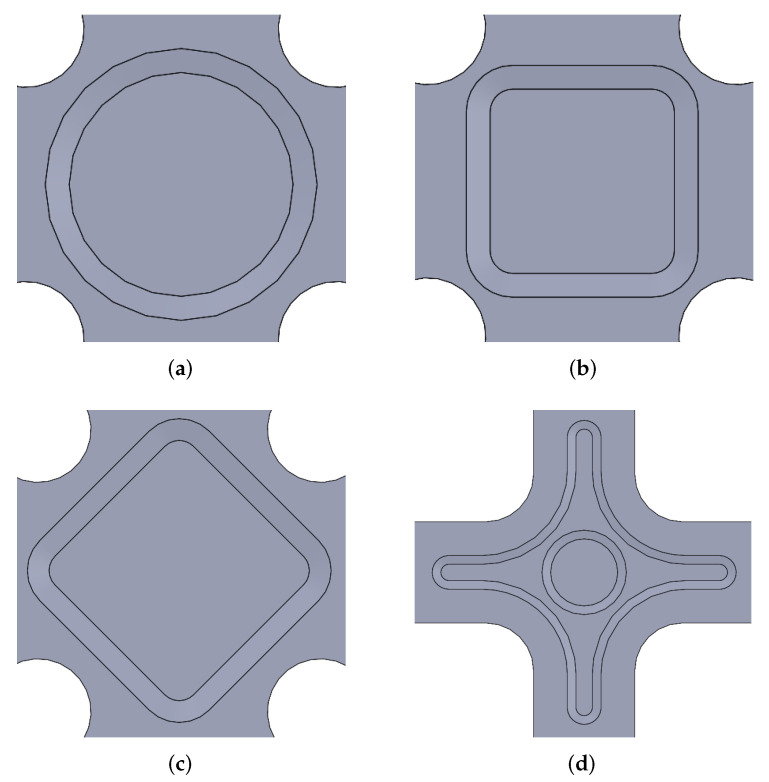
Gauge zone designs frequently found in literature: (**a**) Squared central zone. (**b**) Circular central zone. (**c**) Rhomboidal central zone. (**d**) Double tapering.

Nevertheless, Ramault et al. [61] demonstrated that similar test specimens reinforced with tabs resulted in lower values of failure loads, compared with numerically controlled (CNC) machined thickness tapering, due to the debonding of the tabs. Therefore, the milling option is preferable, although damage generated by machining should be minimised. In this way, starting from a laminate with a higher number of layers than the one to be studied, the external layers are removed in the gauge zone. It should be noted that the external layers may be composed of a stacking sequence similar to the one sought to be analysed in the biaxial zone, or the use of layers with other orientations can be proposed in order to increase the strength out of the gauge region. The CNC pocket machining approach is applied in a vast majority of biaxial tests with cruciform specimens, while the greatest discussion arises in the shape and dimensions that the tapered zone should have. As a summary, Table 2 lists the options chosen in literature consulted in this review after different numerical and/or experimental studies, while Figure 3a–c depicts these possibilities. It is verified that there is no agreed-upon option, although it should be noted that the great variety of existing FRP materials and the important influence of their anisotropic behaviour lead authors to believe that there is not a unique solution to this issue.

An interesting exception to the general trend shown in Table 2 are the works of Yu et al. [122] and Batista [123], which present a double tapered thickness specimen (represented in Figure 3d). Specifically, in the work of Batista, several geometries are analysed for both thickness reductions. The largest taper is produced in the central area, with circular and rhomboidal shapes, while a minor thickness reduction extends to a large part of the specimen arms. After different simulations using FEM, a circular central tapering is chosen, carrying out experimental tests that are collected in [47].

**Figure 4:**
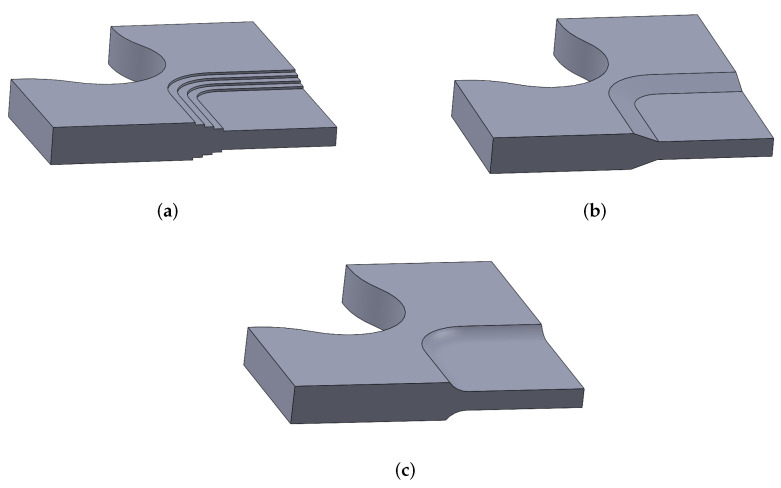
3D model of a quarter of cruciform specimen presenting different techniques for thickness tapering: (**a**) Stepped transition. (**b**) Linear reduction. (**c**) Curved transition.

Although different thickness transitions are observed in the literature (some of them represented in Figure 4), whether they are carried out in the machining of the characterised material itself or in the protecting tabs, few studies analyse in depth the effect of this geometric feature. Specifically, Baptista et al. [98], in the optimisation of the cruciform geometry by applying a direct multi-search algorithm, establish as one of the nine design variables the exit angle of the spline that forms the cross section of the central tapering. The result of this study provides values of angles dependent on the thickness of the arms of the specimen, presenting a tendency to need smaller angles as the thickness increases. However, the study concludes in the need to continue investigating this variable since it is necessary to ensure a correct geometric transition between the maximum and minimum thicknesses that avoids stress concentration. Creuziger et al. [121] carried out a similar study using a thickness transition similar to the one represented in Figure 4c. The researchers performed an optimisation of a metallic cruciform specimen by means of the parametrisation of the dimensions of the central zone pocket, including the radius of the curved thickness transition. They concluded that, on the one hand, this radius should be as high as possible in order to avoid outer stresses larger than the stresses in the gauge zone. On the other hand, this radius is limited due to manufacturing constraints. Finally, Kobeissi et al. [85] investigated the machining of aluminium tabs with linear and curved transitions, concluding that the curved transition could postpone the failure outside the gauge zone.

Despite all of the above, other studies proposed specific approaches for certain materials or load states. For example, Serna Moreno et al. [39,106] defended the need to vary the dimensions of the arms and central thickness tapering depending on the applied loading ratio in perpendicular directions. It should be noted that this aspect becomes even more relevant in laminates composed of unidirectional plies, where the orientation of the fibre in the different layers can give rise to an entirely different apparent response for both loading directions, although the literature dealing with this line of research is scarce [78]. On the contrary, Moncy [46] stated that it is better not to vary the geometry with the load state in order to avoid specimen-related uncertainties, although the author also indicated that it is necessary to take into account in the specimen design the material anisotropy (i.e., different stacking sequences), different load ratios and geometric parameters. Accordingly, numerical simulations carried out by Horta Muñoz and Serna Moreno et al. [40,78,107] demonstrated how the tensile-compressive stress state favours the strain concentration in the biaxially loaded region, opposite to what is observed in equibiaxial tensile or compressive loading.

Other works underline the significant influence of the thickness ratio between the central zone and arms, being noteworthy the work of Baptista et al. [98], in which the previously mentioned optimisation variables are dimensionless compared to the thickness of the arms. This influence is further significant in tests including compressive loads. In this way, Serna Moreno and Horta Muñoz numerically analysed the influence on the critical buckling load and the buckling mode in specimens with different thickness ratios [82]. The authors concluded that certain thicknesses ratios significantly affect the buckling load, and a change in buckling mode shape is expected. Recently, Giannella et al. [124] performed an analogous study, that is, numerically studying the stability of two different geometries of cruciform specimens under compressive loading, in this case, aiming for fracture characterisation. Authors agree on the special influence of the thickness of the central region, pointing out the contrast in the selection of this parameter, as it should be high enough to avoid instabilities, but small enough to guarantee high stress values in the biaxially loaded zone. Another solution to the problem of instability in slender arms of cruciform specimen is given similarly in the works by Manshadi et al. [75,76] and Xu et al. [45], removing the arms of the cruciform specimen and applying the compressive loadings directly over the central region. However, this could lead to increase stress concentrations close to the gauge zone.

**Figure 5:**
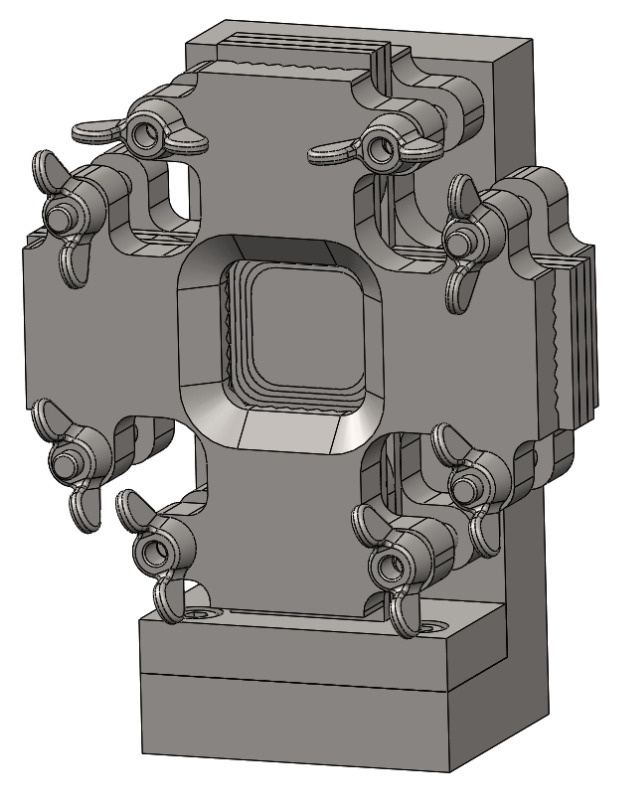
3D model of the anti-buckling device developed by Serna Moreno and Horta Muñoz [82].

If arms are kept in the design of the specimen, some works recommend the application of an anti-buckling device under compressive loading, which usually are designed ad hoc for each combination of biaxial specimen and testing facility [41,44,58,82,83]. As a distinguishing characteristic, it is found that some of these fixtures completely prevent the displacement perpendicular to the loading plane, while other designs comprise openings that allow recording the state of strains in the ROI. As a matter of example, Figure 5 shows the biaxial anti-buckling device designed by Serna Moreno and Horta Muñoz [82], which includes a central window allowing the measurement by means of DIC in the central region of the specimen. The authors emphasised that this device, apart from helping to postpone the buckling of the specimen, facilitates the alignment of the loading system, a characteristic which is especially relevant when compressive loads are applied.

## 4. Novel Methodologies Based on Cruciform Biaxial Testing

This section includes some cutting-edge innovations made on the cruciform biaxial test, as well as methods that, even though are based on the fundamentals of cruciform biaxial testing, differ significantly from that previously described. Although not all of them are currently applied to FRP, they present results that are interesting for future studies on this material field.

Multi- and microscale simulations provide a numerical approach that can give further insight into damage initiation mechanisms at the constituent level in composite materials [125]. Feedback from biaxial tests and micromechanical simulations can be envisaged. On the one hand, experimental tests provide a valuable source of data for the calibration and validation of numerical models that attempt to predict multiaxial failure by means of microscale simulations [126,127]. On the other hand, multiscale simulations can help to better understand experimental testing, as well as assessing specimen design. This last aspect is still underdeveloped in composites, but there is an interesting work on metals by Luo et al. [128]. In this work, a microscale damage criterion that incorporates crystal plasticity is applied to predict fatigue crack initiation and propagation, which is then reproduced in an experimental biaxial test.

In the experimental field, there are also studies that pursue biaxial characterisation at the microscale level. The work carried out within the framework of the ERC-MULTIAX (multiaxial and multiscale plasticity in metals) project, although oriented to the plasticity of metals, can provide the basis for similar work in composite materials. In particular, one of their works [129] describes the design of a biaxial testing machine capable of applying loads on specimens with dimensions on the millimetre scale such that this loading frame can be used inside a scanning electron microscope (SEM) chamber. The biaxial rig consists of four piezoelectric actuators, with independent load cells with a capacity of 44 N and displacement resolutions of the order of 10 nm, both in tension and compression. In addition, another study by this same group of researchers [130] described the microscopic characterisation of a biaxial tests applying technologies such as in situ synchrotron X-ray diffraction and DIC applied to SEM images. Although it is possible to find studies with similar material monitoring techniques applied to FRP composites during uniaxial testing (for example, [131]), to the knowledge of the authors, there are currently no studies that combine the versatility of cruciform tests to generate different loading ratios and the observation of their effects at microscopic levels.

Some researchers [120,132] have studied the influence of combining impact loads and biaxial plane stress states, taking into account that their effects appear simultaneously in real composite structures, such as in aircraft or wind turbines. Specifically, Kumazawa et al. [120] analysed the simultaneous combination of in-plane static and transverse dynamic loads. For this purpose, they applied a high velocity impact with a steel ball on previously uni- and biaxially loaded specimens in order to study crack propagation in the presence of these multi-axial loading states. Jia et al. [132] proposed a scheme similar to that followed in the compression after impact (CAI) tests [133], applying firstly a low-velocity impact and subsequently evaluating the residual static strength but in a tensile–tensile biaxial test.

Milner and Gnäuper-Herold [89] proposed an innovative cruciform specimen design, trying to respond to two of the main issues of cruciform specimens. On the one hand, they sought to improve the homogeneity in the stress/strain state obtained in the gauge zone, while on the other hand, they tried to avoid premature failure usually produced outside the biaxially loaded zone. To do this, instead of making changes to aspects usually analysed (i.e., geometry of the corners, thickness tapering, and slotting arms), they devised a specimen formed by eight loading arms, called an “Octo-Strain” specimen. In order to test this specimen, it was also necessary to develop a new test facility consisting of eight electromechanical actuators. In addition, advanced technologies such as neutron diffraction and DIC were applied, the last one in order to achieve a strain-based control scheme. The authors of this study concluded, based on the experimental evidence, that the Octo-Strain specimen exceeds the results obtained with traditional cruciform specimens, reaching maximum strains twice as high as those achieved with an equivalent four-armed biaxial specimen.

The work of Androulidakis et al. [134] focused on the study of the mechanical behaviour of a graphene sheet in a state of biaxial strain. The authors’ approach is very striking: the need to test a nanometric-thick material to which a biaxial load cannot be applied directly, which led the authors to produce this graphene sheet on a polymer cruciform specimen. To control the applied state on the graphene sheet, the deformation is not produced by a flat stress state, but by applying a load perpendicular to the plane. This produces the bending of the specimen, obtaining the maximum tension or compression strains in the graphene sheet.

Several authors have delved into the simultaneous application of cryogenic temperatures to biaxial tests with cruciform specimens, whose main application lies in the study of composite materials for aerospace use in cryogenic propellent tanks. Among other studies, French et al. [135,136] combined the application of non-linear numerical simulations and experimental testing, including the use of X-ray computed tomography (CT), to analyse the evolution of ply-level damage in laminates with different ply orientations under the so-called cryobiaxial loads. Specifically, three specimen designs were proposed and analysed, based on different methods of thickness tapering, highlighting one design that uses tabs with a sharp corner transition that restricts the gauge region to the diagonals of the central zone of the specimen. In this way, a small region is created, but with a high concentration of tensile stress across the diagonals of the gauge zone, promoting the appearance of vertically transverse cracks. The combination of instrumentation during the test by means of 3D-DIC and ex situ measurement of damage evolution by X-ray CT allows direct comparison with progressive damage analysis preformed in a numerical software.

In addition, focusing on FRPs used in propellant tanks for space vehicles, Hamori et al. [137] investigated the appearance of matrix cracking under biaxial loading states that may allow fuel leakage. In order to reduce the damage caused to the material, or at least to mitigate the gas loss, the use of CFRP thin-plies acting as barrier layers is proposed. To do this, four layers oriented in a cross-ply arrangement are introduced in a laminate in which the apparent thickness of the main layers is four times greater than that of the barrier layers. The experimental tests carried out at temperatures of $-253{\;}^{\circ }$C show how the specimens that have thin-ply barrier layers allow a higher level of deformation to be achieved without leaks. In addition, the study demonstrates through numerical simulations how the microcracks permitting gas leaks are formed, qualitatively validating the conclusions of the experimental campaign.

Outside the domain of FRP for structural application, biaxial tests on cruciform specimens have become a frequent option for the characterisation of materials with biomedical applications. One of the most notable utilisations of these tests in the medical field is the characterisation of soft tissues, as these usually behave as membranes submitted to plane stress. For instance, Jiang et al. [138] developed a test system capable of applying both tensile and simple shear loads, detailing the development of the control system and the methodology applied for data acquisition. Additive manufacturing (AM) technologies are evolving towards the manufacture of fibrillar composite materials, with characteristics close to real natural tissue. In this sense, Page et al. [139,140] developed tissue scaffolds based on the ideas of angle-ply fibre laminates, highlighting the need for a micromechanical constitutive behaviour, which allows the regeneration of biological tissues. Moreover, the application of FDM is also interesting to develop elements that can be used in wearable medical devices and present anisotropic behaviour that must be characterised by means of multiaxial testing [141].

Following with the application of AM technologies, it is expected to see an increase in the use of FRP obtained through techniques such as FDM in next years. Although it is possible to find numerous works investigating the behaviour of these materials under different loading states, today, it is still difficult to find works that analyse their multiaxial response. However, this is highly recommendable, as these are materials with a high anisotropy, due to both heterogeneity and to the fused deposition process. Kang et al. [112] carried out one of the first biaxial studies on this type of material, specifically testing polyether ether ketone (PEEK) reinforced with short carbon fibre. Through a methodology that combines uniaxial tests with a numerical model that includes anisotropy and plasticity of the material, the researchers determined the stress–strain response of the material subjected to five different stress ratios.

Finally, fracture toughness tests are of great interest in the characterisation of materials. These are usually performed by uniaxial tensile loads and bending loads on specimens with geometries that favour crack initiation in the fracture modes of interest. Nevertheless, biaxial tests on cruciform specimens are also gradually being adapted for this purpose, once again due to the more realistic stress states obtained. Currently, most advances in this field occur in metals [103,142,143], although fracture studies under biaxial loads are beginning to appear in composites, where the brittleness of the material leads to the appearance of microcracks whose uncontrolled evolution can result in catastrophic failure. For example, Dang et al. [144] demonstrated the importance of the experimental characterisation of materials with pre-induced cracks under biaxial loading, where the strengthening effect detailed in other studies [35,37,78,119] is also present. Tests were carried out with different orientations of the initial crack ($0^{\circ }$ and $45^{\circ }$), and the results are compared with numerical models of crack initiation and propagation. Authors conclude that the laminate supports higher loads than those predicted from uniaxial states; therefore, safe designs can be made with safety factors lower than those obtained by traditional characterisation. From the numerical point of view, the work of Navarro-Zafra et al. [145] investigated the application of the extended finite element method (XFEM) to the evaluation of the stress intensity factors under biaxial loading, reproducing the crack initiation and propagation in various short GFRP cruciforms specimens submitted to different applied forces. Simulations reproduced a mode I dominated failure, which corresponds with previous experimental evidences. The same authors [146] delved into modelling the non-linear response of the material using a 3D progressive damage model based on mixed failure modes (fibre rupture and matrix cracking). Numerical results are obtained in terms of dissipated energy and failure initiation stresses, showing good agreement with the experimental data.

## Figures and Tables

**Table 1:** Preferred shape for corner between adjacent arms in experimental biaxial tests with cruciform specimen.

Shape	References
Single fillet radius	[42,50,74,81,83,92,93,104,105]
Double fillet radii	[16,35,36,37,38,39,40,43,46,55,63,64,71,80,94,95,106,107,108,109,110,111,112]
Elliptical fillet	[17,65,68,86,98,113,114]
Slotted arms	[33,74,79,112,115,116,117,118,119]

**Table 2:** Preferred shape for central gauge zone in experimental biaxial tests with FRP composites.

Shape	References
Circle	[42,43,47,74,81,83,93,94,95,98,112,121]
Rhombus	[50,77,104]
Square	[16,17,35,36,37,38,39,40,45,63,64,65,106,107,108,111,115,119]

## Data Availability

Not applicable.
